# The provision and utilization of essential health services in Afghanistan during COVID-19 pandemic

**DOI:** 10.3389/fpubh.2022.1097680

**Published:** 2023-01-12

**Authors:** Narges Neyazi, Christina Lindan, Saber Perdes, Abdul Ghani Ibrahimi, Dirk Horemans, Deena Al Afsoor

**Affiliations:** ^1^Health System Development Department, World Health Organization, Kabul, Afghanistan; ^2^Department of Epidemiology and Biostatistics, and Institute of Global Health Sciences, University of California, San Francisco, San Francisco, CA, United States; ^3^Nezarat Consulting Ltd., Ottawa, ON, Canada; ^4^Department of Universal Health Coverage, World Health Organization, Geneva, Switzerland; ^5^Department of Universal Health Coverage, Eastern Mediterranean Regional Office, World Health Organization, Cairo, Egypt

**Keywords:** Afghanistan, COVID-19 pandemic, maintaining, essential, health services, resilience, health system, recovery

## Abstract

**Introduction:**

The COVID-19 pandemic has disrupted provision of essential health services and overwhelmed even robust health systems worldwide. The Afghanistan health system has suffered both from the pandemic, as well as from political upheaval and regime change.

**Methods:**

We evaluated essential service delivery using data collected from a cross-sectional survey of health care facilities in Afghanistan based on administration of a World Health Organization standardized assessment of frontline service readiness. A multi-stage sampling scheme was used to identify a representative sample of 92 health facilities (68 clinics and 24 hospitals) providing essential health services in five provinces. Facility managers were asked to report on changes in health service delivery in late 2021 and early 2022 (corresponding to the end of a significant national COVID-19 surge in infections) compared to the same period one year earlier.

**Results:**

Among health facilities evaluated; 29 were in urban and 63 were in rural settings. Most facilities reported an increase in the provision of outpatient care particularly in maternal and child health services as well as for tuberculosis, chronic respiratory diseases, mental health, and substance abuse; the number of in-patients also increased. In contrast, provision of services for malaria, neglected tropical diseases, and community outreach programs decreased. Nearly all facilities used strategies to maintain services, including targeting high-risk patients, promoting self-care, and redirecting patients to alternative health care sites. Nearly three fourth (70.6%) of facilities provided no training about COVID-19 to staff; only 65.2% referred COVID-19 patients to designated hospitals and 44.6% had safe transportation for these patients.

**Discussion:**

Increased demand for services during this period was likely due to a backlog in need generated during the preceding COVID-19 surge and the political changes happened a few months earlier to this survey. Facilities used various methods to maintain services, although the decrease in provision of community outreach was concerning. Facilities appeared to be able to maintain essential health services, despite an increase in demand. However, awareness and training of COVID-19 protocols and appropriate and safe referrals need to be improved. In general, these series of surveys are informative and helpful to identify any changes in provision of essential health services and can facilitate recovery of health systems during and after pandemics.

## Introduction

The global spread of SARS-CoV-2 infections (COVID-19) was declared a public health emergency by the World Health Organization (WHO) in early 2020 (1). As of November 2022, more than 632 million infections and 6.6 million deaths have been reported globally (2), an underestimate of the true burden of disease due to limited access to testing and surveillance in many countries. The pandemic has challenged public health systems worldwide, revealing that even seemingly robust health systems can be rapidly overwhelmed and compromised (3–6). Health seeking behavior for routine care also declined during the COVID-19 pandemic (7–10). In the US, for example, the combination of increased workload and reduced number of health workers due to infection, fears about exposure, and burn-out, led to a severe strain on the capacity to maintain essential services (11). Mortality related to disruption in essential health service delivery during an epidemic can exceed the number of deaths directly attributed to the disease itself (12, 13). Although the impact of the COVID-19 pandemic has been well-characterized in the US and Europe, less is known about what occurred in Lower Income Countries (LICs), particularly in areas that also experienced political upheaval, such as Afghanistan.

**Figure 1:**
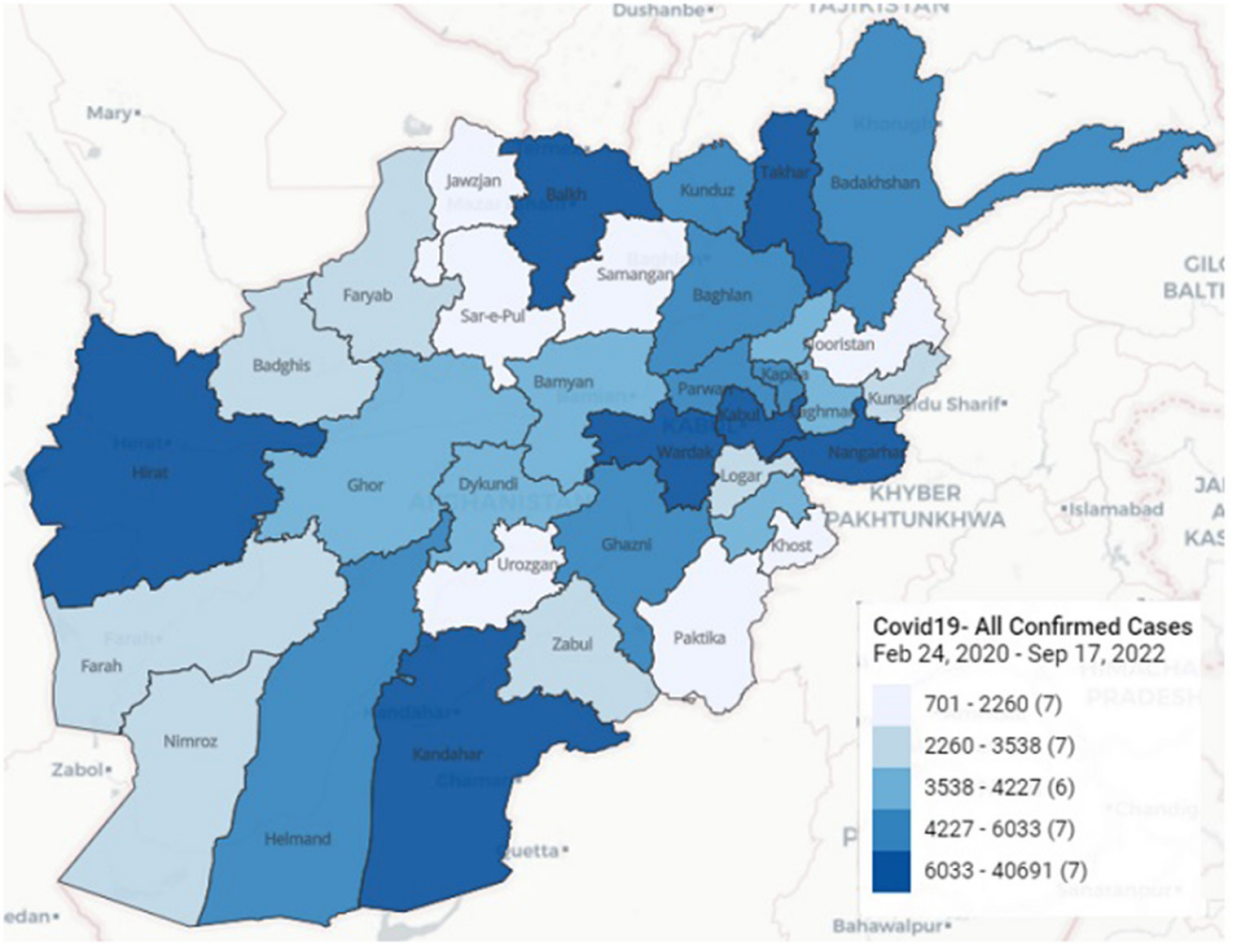
Geographical distribution of COVID-19 confirmed cases in Afghanistan (24 Feb 2020-17 Sep 2022) (48).

**Figure 2:**
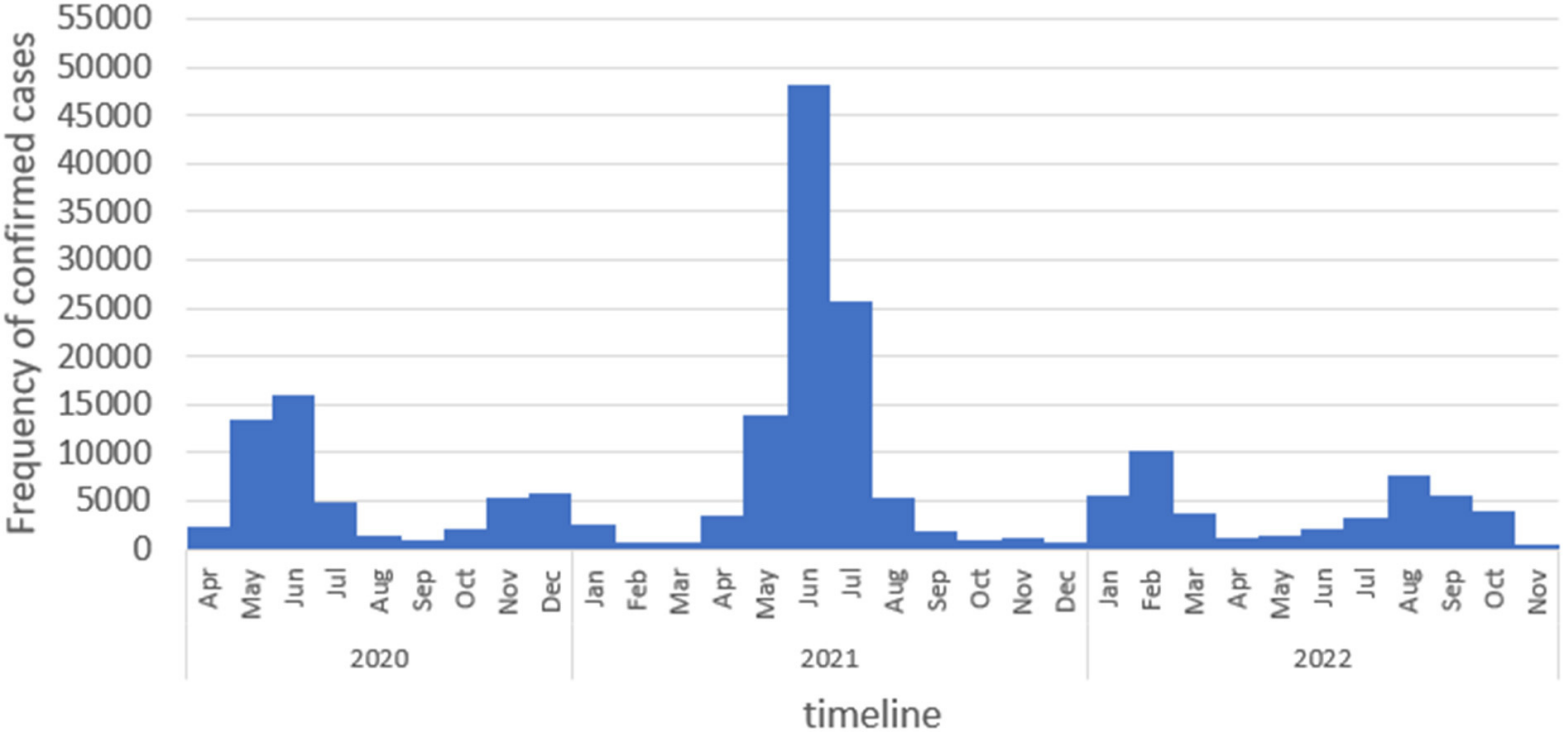
Frequency distribution of COVID-19 positive cases in Afghanistan. April 2020 to Nov 2022. Source: DHIS2, Afghanistan Ministry of Public Health.

The first case of COVID-19 in Afghanistan was identified in February 2020 in Herat Province, in the west of the country. By November 2022, more than 200,000 confirmed infections and nearly 8,000 deaths had been reported (14). Nearly half of all cases were detected in the five most populous provinces (Kabul, Herat, Kandahar, Balkh, and Nangarhar) (Figure 1). Afghanistan has experienced five surges of COVID-19 since early 2020 (Figure 2). At the start of pandemic, the Afghanistan Ministry of Public Health (MoPH) designated specific hospitals, at least one in each of the 34 provinces, to take care of COVID-19 patients, leaving other health facilities to continue to provide routine and essential care. People with clinical or laboratory-confirmed infection were to be referred to the COVID-19 hospitals. The National Disease Surveillance and Response (NDSR) report shows that there is an increase in trend of Pneumonia and Measles incidence percentage over the past 3 years in Afghanistan (15).

Policy decisions to mitigate health system bottlenecks during any pandemic, including COVID-19, should be informed by accurate and real-time data collected through ongoing tracking and monitoring of health services. To obtain a rapid snapshot of changes and challenges in service delivery and utilization, the WHO designed national “pulse surveys” that could be implemented to help evaluate the continuity of essential health services and identify critical bottlenecks during the pandemic (16). Several of these surveys were implemented in Afghanistan, the results of which suggested that 65% of essential health services in early 2020, and 10% of these services in early 2021, had been disrupted (17). The periods during which the surveys were administered, coincided with the first three waves of COVID-19 in the country.

The pulse surveys were designed to be administered to key informants or senior program managers, often at the national level, with responses based on their own assessments; therefore, the surveys did not obtain information from individual health facilities themselves and data were subjective. Therefore, the WHO developed a suite of frontline service readiness surveys in 2021 to measure the extent to which facilities provided essential health services and COVID-19 case management during the pandemic; a component of the surveys also assessed community needs (18). These WHO surveys were implemented in 18 countries in sub-Saharan and north Africa, eastern Europe, and Latin America at various times during 2021–2022 (19–37). In this current paper, we present the results of the first round of this frontline service readiness survey administered in Afghanistan to a representative sample of non-COVID designated health facilities from five provinces. The timing of the survey corresponded to the end of third wave of COVID-19 in the country.

## Materials and methods

### Summary

We present the results of a survey administered to health facility mangers of 92 health facilities to evaluate self-reported changes in health services following the third COVID-19 surge in Afghanistan. This survey was developed by the WHO (18) and modified for local use. We used a multi-stage sampling scheme to identify a representative sample of health facilities, both hospitals and clinics in five provinces, not including facilities that were specifically designated to provide COVID-19 care. Data collection was conducted in February 2022; facility managers were asked to report on changes in health service delivery from November 2021 through January 2022 compared to the same 3 months a year earlier.

### Study population and sampling

We selected five of the seven regions in Afghanistan; within each of the five regions, we selected the most populous province. In 2019, 15.5% of the total country population was estimated to reside in these five provinces (38), which include 81 districts. We randomly selected 20 districts, in which 222 health facilities and 29 non-COVID hospitals were operating. We randomly selected 63 primary healthcare facilities and included all 29 non-COVID designated hospitals in the sample. Some clinics managers (*N* = 17) were not available for the interview, and were replaced by identifying another randomly selected site. All managers of selected hospitals were available.

### Measurements

The standardized WHO questionnaire “Continuity of Essential Health Services: Facility Assessment Tool” (39) was used for this study. The questionnaire was content validated by the WHO country office and the Afghanistan MoPH. The survey was piloted among five managers of health facilities not included in the sampling frame and questions were adapted to the Afghan context prior to administration. The questionnaire was administered to facility managers by trained staff of the Afghanistan National Public Health Institute (ANPHI), by phone from 8 to 20 February 2022. Responses were entered electronically using the offline LimeSurvey application (40) and uploaded to a secure database. The questionnaire included 169 questions and took ~80 min to complete. In this paper, we only report on a subset of the data collected, and do not include information collected from community leaders using a different component of the parent survey.

We evaluated whether the health care facility was managed by the government or by a non-public/non-governmental local organization (NGO), was situated in an urban or rural area, whether it provided only out-patient services or included in-patient care, the number of in-patient beds, and the number of health care and non-health care staff. Questions were asked about the following in the previous 3 months: the number of staff who became infected with SARS-CoV-2; whether new staff numbers or re-allocation of staff were required to accommodate patient load; and whether training about COVID-19 was provided. These trainings include infection prevention and control (IPC), proper use of personal protective equipment (PPE), triage protocols for COVID-19 case management, management of emergency conditions, and provision of remote health care.

Questions were asked about changes in last 3 months in service delivery and utilization of different types of outpatient services, emergency unit visits for non-COVID-19 related issues, provision of outreach services, and inpatient admissions. The survey also asked about changes made to control the spread of COVID-19, to maintain the essential service delivery and whether facilities could refer infected patients to COVID-19 designated treatment centers.

### Statistical analyses

Data were entered into excel, cleaned, and then exported into and analyzed using Stata version 17. We calculated the frequency distribution of characteristics of facilities, and responses to other questions, stratified by urban and rural facility.

### Ethical considerations

The study protocol was reviewed and approved by the ANPHI of the MoPH institutional review board (IRB Code No: A.0122.0389). Verbal informed consent was obtained from each facility representative who was interviewed.

## Results

Of the 92 health facilities evaluated, 29 were in urban and 63 were in rural settings; 77.2% of them were managed by local NGOs (Table 1). The median (IQR) number of beds in facilities (*N* = 51) was 10 (6–49) in rural facilities and 40 (8–222) in urban facilities. More than half of all staff (64.7%) were clinical. Staffing of urban facilities (median 212.4 staff/facility) was much higher than for rural facilities (17.1 staff /facility). Overall, 70.6% of facilities received no COVID-19 related training in the past 3 months; 34.5% of urban compared to 7.9% of rural facilities received training in all five topics. Approximately a tenth of all staff (12.0%) were diagnosed with COVID-19 in past 3 months; 29.3% of facilities had to increase or re-direct staffing to accommodate changes in the volume of patients.

**Table 1:** Characteristics of health care facilities and changes during November 2021-January 2022, Afghanistan (*N* = 92).

**Characteristic**	**All (*****N*** = **92)**		**Urban (*****N*** = **29)**		**Rural (*****N*** = **63)**	
	***N***	**(%)**	***N***	**(%)**	***N***	**(%)**
**Management**						
Government	21	22.8	12	41.4	9	14.3
NGO	71	77.2	17	58.6	54	85.7
**Service provided**						
Only outpatient	40	44.0	4	13.8	36	58.0
Outpatient and inpatient	51	56.0	25	86.2	26	42.0
Inpatient beds/facility, median (IQR) (*N* = 51)^a^	10	(10–49)	40	(8–222)	10	(6–20)
**Number of staff**	***N*** **= 7,240**		***N*** **= 6,161**		***N*** **= 1,079**	
Clinical	4,687	64.7	3,936	63.8	751	69.6
Non- clinical	2,553	35.3	2,225	36.2	328	30.4
Average number of staff / facilities	78.6		212.4		17.1	
**COVID-19-related training topics provided, last 3 months** ^b^						
5 topics	15	16.3	10	34.5	5	7.9
3–4 topics	3	3.3	1	3.4	2	3.2
1–2 topics	9	9.8	5	17.2	4	6.3
No training	65	70.6	13	44.8	52	82.5
**Referral of COVID-19 patients**						
Aware of COVID-19-specific hospitals	60	65.2	23	79.3	37	58.7
Safe transportation for referral	41	44.6	21	72.4	20	31.7
**Impact of COVID-19 on staff, last 3 months**						
Staff diagnosed with COVID-19	868/7,240	12.0	800/6,161	13.0	97/1,079	9.0
Facilities requiring increased or change in staffing to accommodate patient volume or patient type related to COVID-19^c^	27	29.3	10	34.5	17	27.0

a 51 facilities had inpatient beds (excluding those used for delivery).

b Topics included: infection control, use of PPE, triage of COVID-19 patients, management of emergency conditions, remote health care.

c Changes related to patient/volume type because of COVID-19, including reassignment, increasing hours or overtime, new staff recruitment, use of volunteers, switch to different facility, layoff or unpaid leave.

Eighty-eight health facilities reported providing outpatient services, of which 88.6% reported an increase in provision of outpatient services during Nov 2021-Jan 2022 (Table 2). Most reported increased delivery of family planning (73.0%), antenatal (79.0%), postnatal care (75.0%) and pediatric care (79.3%), and immunization (68.7%). For infectious and non-communicable diseases, facilities reported the highest increase in service delivery and utilization for tuberculosis (64.3%), chronic respiratory diseases (76.5%), mental health and substance abuse (69.3%). Only a small proportion of facilities reported a decrease in services, mostly for malaria (25.9%), neglected tropical diseases (24.6%), and intimate partner and sexual violence (30.7%). Among the 78 facilities that reported an increase in outpatient services, the main reason provided was addressing backlog from disruptions of services prior to the past 3 months (Data not shown). Among the eight facilities (9%) that reported a decrease in provision of outpatient care, the main reason for the change was disruption in ability to provide services including limited availability of medicines or consumables and limited availability of medical staff (data not shown).

**Table 2:** Number of facilities with self-reported changes in service delivery and utilization, November 2021-Janurary 2022, compared to a year previously, Afghanistan.

	**Change in service delivery**					
**Type of service**	**Increased**	**Decreased**	**No change**			
	***N***	**%**	***N***	**%**	***N***	**%**
**Outpatient**						
Any (*N* = 88)^a^	78	88.6	8	9.0	2	2.3
Non-specific symptoms^b^ (*N* = 89)	77	86.5	5	5.6	7	7.8
**Family planning/ante and prenatal care, pediatrics, immunization**						
Family planning, contraception (*N* = 89)	65	73.0	10	11.2	14	15.7
Antenatal care (*N* = 89)	71	79.8	4	4.5	14	15.7
Postnatal care (*N* = 88)	66	75.0	6	6.8	16	18.2
Immunization (*N* = 83)	57	68.7	10	12.0	16	19.3
Pediatrics (*N* = 87)	69	79.3	5	5.7	13	15.0
**Infectious disease**						
HIV (*N* = 59)	21	35.6	8	13.6	30	50.8
Tuberculosis (*N* = 70)	45	64.3	10	14.3	15	21.4
Malaria (*N* = 81)	23	28.4	21	25.9	37	45.7
Neglected tropical diseases (*N* = 73)	27	37.0	18	24.6	28	38.4
Sexually transmitted infections (*N* = 73)	21	28.8	9	12.3	43	58.9
**Non-communicable disease**						
Chronic cardiovascular disease (*N* = 62)	25	40.3	9	14.5	28	45.2
Chronic respiratory disease (*N* = 81)	62	76.5	5	6.2	14	17.3
Diabetes (*N* = 64)	24	37.5	7	11.0	33	51.6
Cancer (*N* = 35)	11	31.4	4	11.4	20	57.1
Mental health, substance abuse (*N* = 75)	52	69.3	10	13.3	13	17.3
Intimate partner or sexual violence (*N* = 65)	18	27.7	20	30.7	27	41.5
Physical rehabilitation (*N* = 68)	24	35.3	15	22.0	29	42.6
**Emergency unit visits**						
Any (*N* = 55)	26	47.3	14	25.4	15	27.3
Injuries (*N* = 54)	17	31.5	26	48.1	11	20.4
Emergency surgery, including C- section (*N* = 38)	21	55.3	9	23.7	8	21.0
Non-communicable diseases^c^ (*N* = 49)	15	30.6	13	26.5	21	42.9
Blood transfusion (*N* = 48)	21	43.7	3	6.3	24	50.0
**Outreach services**						
Immunization (*N* = 53)	25	47.1	18	34.0	10	18.9
Malaria prevention (*N* = 34)	4	11.8	13	38.2	17	50.0
Neglected tropical diseases^d^ (*N* = 43)	15	34.9	14	32.6	14	32.5
Community-based mobile clinics (*N* = 33)	10	30.0	11	33.3	12	36.4
Home visits (*N* = 41)	18	43.9	10	24.4	13	31.7
**Inpatient admissions (*****N*** = **50)**	34	68.0	7	14.0	9	18.0

a The number in parentheses refers to the number of facilities that provided specific services.

b Fever, pain, fatigue, and cough, not ascribed to another cause.

c Myocardial infarction, arrhythmia, stroke, diabetic ketoacidosis, asthma, chronic obstructive pulmonary disease, and cancer.

d Includes mass drug administration.

We asked the health facilities' managers about the changes in visits from emergency unit for non-COVID-19 related issues. Overall, 47.3% of health facilities reported increase in service delivery and utilization. 55.3% reported an increase in emergency surgery, including emergency Cesarean-section (C-section), followed by a rise in urgent blood transfusion services (43.7%). However, 48.1% facilities reported a decrease in delivery and utilization of services for injuries (Table 2).

Almost one-third (32.5%) of the 55 health facilities providing community outreach, reported a decline in provision of outreach services including immunization (34.0%), malaria prevention campaigns (38.2%), neglected tropical diseases (32.6%), and community based mobile clinics (33.3%). Half of health facilities reported no changes in provision of malaria prevention campaigns, but 47.1% of health facilities reported on a rise in provision of home visits (Table 2).

Among the 51 health facilities that provided in-patient care, 68% reported in an increase in the number of admissions in the previous 3 months, compared to the same 3 months the previous year (Table 2).

We also asked the health facility managers about changes in service provision to control the SARS-CoV-2 transmission and strategies used to maintain the provision of essential health services in the period of study (Table 3); 33.0% reduced the scope of specific services and 28.2% reduced number of patients that could be seen. Most health facilities tried to maintain health service delivery using strategies like targeting high-risk patients (95.5%), promoting self-care (93.5%), redirecting patients to alternative healthcare facilities (87.0%), providing all care in a single visit for multiple morbidities (78.8%), and using home-based care (68.5%). The application of these strategies by health facilities was similar in urban and rural areas. Only 65.2% of health facilities reported that there was a referral system for COVID-19 patients, however, 44.6% of these facilities only had access to safe and isolated transportation to transfer the patient's following referral.

**Table 3:** Facilities that modified service delivery during the months November 2021-January 2022, Afghanistan (*N* = 92).

**Service delivery** **modification**		**All (*****N*** = **92)**		**Urban (*****N*** = **29)**		**Rural (*****N*** = **63)**	
		***N***	**(%)**	***N***	**(%)**	***N***	**(%)**
Strategies to control of COVID-19 spread	Closed	4	4.3	3	10.3	1	1.6
	Change in service hours	17	18.5	6	20.7	11	17.4
	Reduced scope of specific services	30	33.0	7	24.1	23	37.1
	Reduced number of patients seen	26	28.2	6	20.7	20	31.7
	Suspended provision of specific services	9	9.8	2	6.9	7	11.1
Strategies to maintain essential health service delivery	Redirected patients	80	87.0	24	82.7	56	88.9
	Targeted high-risk patients	86	95.5	28	100.0	58	93.5
	Provided single visit for multiple morbidities	72	78.8	22	75.8	50	79.2
	Promoted self-care	86	93.5	26	89.6	60	93.5
	Used home-based care	63	68.5	18	62.0	45	71.4
	Used tele-medicine	45	48.9	13	44.8	32	50.8
	Used tele-prescription	29	32.2	11	37.9	18	29.5

## Discussion

In this cross-sectional study of a representative sample of hospitals and clinics from five provinces in Afghanistan, a large proportion of health care sites reported changes in the volume of patients and essential health service delivery over three-months in late 2021 and early 2022, corresponding with the tail-end of the last COVID-19 surge in 2021. Most facilities reported an increase in the provision of outpatient services, particularly in maternal and child health including immunization, family planning, and emergency C-sections. The main reason reported for increased demand was a backlog in request for services. During high levels of community transmission of SARS-CoV-2 immediately prior to the period surveyed, women may have refrained from seeking health care to reduce their exposure to infection. Because we do not have comparison data during the surge or at other time points, we can only rely on responses to the few questions that asked about overall causes of change in demand. Nevertheless, the increase in need for maternal and child health care may indicate a greater toll of the pandemic on delayed access to care among women and children, who are particularly vulnerable.

A time series analysis in 18 low- and middle-income countries including Afghanistan from 2018 to 2021 estimated an average 13.1% decline in outpatient volume and average decline of up to 5% in utilization of maternal and child services (41). These declines were associated with an estimated 3.6 and 1.5% increase in child and maternal mortality, respectively. Because Afghanistan does not have a death registry and data on mortality were further jeopardized by political chaos after the takeover by the Taliban, we do not know whether any of the service delivery changes identified here were associated with increased mortality.

More than half of facilities also reported increase in utilization and delivery of care and treatment of tuberculosis, chronic respiratory diseases, mental health, and substance abuse, and about half reported an increase in emergency department visits. These changes may also have been as a result of previous delays in health seeking. In contrast, almost two-thirds of facilities reported either a decrease or no change in demand for treatment of injuries, which may be due to less political violence during this period.

In our study, nearly half of health facilities reported increasing reliance on home-based care, which can reduce exposure to COVID-19 and be helpful when transportation is restricted. Other countries have also taken a similar approach to reducing transmission by providing non-facility-based care (42–45). The decline in community outreach services including malaria prevention campaigns, neglected tropical diseases, and mobile clinics may have been another method of reducing transmission of SARS-CoV-2. Alternatively, the demand for treatment of malaria and neglected tropical diseases may have decreased due to lower incidence of these infections during cold weather months.

Facilities reported making other changes to service delivery: about one-third reduced the volume of patients and/or changed the type of services that they provided; a much smaller number either closed or discontinued some services. The reasons for these changes cannot be inferred from the questionnaire itself, however. The relatively small proportion who made these changes may have been because of a decline in reported SARS-CoV-2 infections during the months to which the survey referred; alternatively, facilities may not have wanted to make changes despite COVID-19 risk, in order to maintain services. Nearly all facilities used at least some strategies to maintain services, including targeting high-risk patients, promoting self-care, and redirecting patients to alternative healthcare facilities.

Despite the availability of COVID-19 specific hospitals in each province, not all facilities referred patients with clinical or laboratory-confirmed infection. It is not clear whether this is because they were unaware of the availability of designated care facilities or did not refer them for other reasons. Less than half of the facilities reported having safe transportation for COVID-19 patients, and most of these were located in urban settings.

Only one-fourth of facilities provided comprehensive training about COVID-19. With the onset of a pandemic and within a short timeframe, healthcare facilities must ensure that personnel are correctly trained and capable of implementing infection control procedures. In a study of 22 African countries, 42,058 frontline healthcare workers were trained during the first wave of COVID-19 pandemic. The evaluation documented significant short-term improvement but indicated that sustained changes required ongoing supportive supervision and monitoring (46). The results of our survey indicate that training around COVID-19 prevention needs to be improved.

The cause of the changes that we found in service delivery could be due to the pandemic but could also be a result of political upheaval after the collapse of the government in August 2021. Most international donors froze their financial support and may have been the reason for the closure of some of the facilities surveyed. Widespread vulnerability due to high levels of poverty, food insecurity, limited access to safe drinking water and sanitation, as well as natural disasters including earthquakes and droughts have all impacted the wellbeing of the population, coupled with nearly 40 years of chronic conflict (47).

Our study had several additional limitations. First, changes in service delivery were by self-report of managers and were not based on collection or review of actual facility -level data. Therefore, respondents may not have been able to recount what happened during the previous 3 months of the survey compared to the same months last year. Availability of facility-level data would have been limited in any case, due to the general lack of electronic health records. Second, many questions in the survey were non-specific, and did not assess whether changes were directly due to a backlog of need due to COVID-19, to the political situation, to reduce exposure of health care staff and the community to SARS-CoV-2, or were due to other reasons. Importantly, we cannot tell from the survey whether the changes in services did not ensure adequate delivery of care. Including a limited number of more open-ended questions or following up with focus group discussions to obtain more nuanced information would be very helpful to understand the implications of the findings. Third, we only present a sub-set of data from the survey and did not include information about COVID-19 related testing or services, or funding issues. Fourth, although we included a representative sample of government and NGO-run facilities, we did not include private sector facilities; however, public health facilities are primarily responsible for essential health care services. We propose conducting the next round of assessments in more provinces of Afghanistan including public and private health facilities.

Finally, our study focused on facilities in the five most populous provinces; these facilities receive greater support than facilities in less sparsely populated areas. Less- resourced health care sites might experience greater or different disruptions in services.

These surveys can be helpful in monitoring fluctuations in service delivery over time, and if followed up with more detailed interviews, and can assist in determining methods to ensure delivery of essential health services. The use of a standardized questionnaire delivered in multiple settings would ideally allow comparisons across countries and WHO regions. The use of an offline electronic data collection was also useful, particularly in a country such as Afghanistan, without access to stable or high-speed internet.

These types of assessments could be used in similar outbreaks or pandemics in future. Information can be used to update the country response plans and development of policies and planning for emergency management within wider efforts to strengthen the country's health system. We propose the following actions: identifying and mapping existing resources and weaknesses to determine priority needs; strengthening competencies of public health professional and their role in emergency management; developing health workforce capacity to engage the local population; adapting policies and planning with monitoring and accountability; determine the needs for long-term health system strengthening to maintain essential health and social services especially for non-communicable diseases, mental health and health emergency preparedness.

Our study showed increase in demand and utilization of many essential health services during the COVID-19 pandemic. After nearly 3 years of the COVID-19 pandemic, Afghanistan should focus more on maintaining essential health service delivery especially for the dual burden of communicable and non-communicable diseases. Control and case management of COVID-19 should be integrated into primary, secondary, and tertiary levels of health system.

## Data availability statement

The datasets presented in this study can be found in online repositories. The names of the repository/repositories and accession number(s) can be found in the article/supplementary material.

## Author contributions

NN, DH, DA, and AI designed the study. NN lead the data collection and analysis of the data. CL, SP, and NN wrote the manuscript. All authors reviewed the manuscript and contributed to its development and approved the final version.
